#  Some New Inequalities of Jordan Type for Sine

**DOI:** 10.1155/2013/834159

**Published:** 2013-10-31

**Authors:** Shan-Peng Zeng, Yue-Sheng Wu

**Affiliations:** ^1^Department of Mathematics, Hangzhou Electronic Information Vocational School, Hangzhou, Zhejiang 310021, China; ^2^College of Science, China Jiliang University, Hangzhou, Zhejiang 310018, China

## Abstract

The authors find some new inequalities of Jordan type for the sine function. These newly established inequalities are of new form and are applied to deduce some known results.

## 1. Introduction

For *x* ∈ (0, *π*/2], we have

(1) $$\begin{array}{c} \frac{\sin x}{x}\geq \frac{2}{\pi }. \end{array}$$

The inequality is sharp with equality if and only if *x* = *π*/2. This inequality is known in the literature as Jordan's inequality for the sine function. See [1, page 33] and other references cited in the first page of [2]. 

In [3, 4], it was obtained that

(2) $$\begin{array}{c} \frac{1}{\pi^{3}}\left(\pi^{2}-4x^{2}\right)+\frac{2}{\pi }\leq \frac{\sin x}{x}\leq \frac{2}{\pi }+\frac{\pi -2}{\pi^{3}}\left(\pi^{2}-4x^{2}\right), \end{array}$$

for *x* ∈ (0, *π*/2]. The equalities hold if and only if *x* = *π*/2. This refines Jordan's inequality (1).

Motivated by [3], it was established in [5] that

(3) $$\begin{array}{c} \frac{1}{2\pi^{5}}\left(\pi^{4}-16x^{4}\right)+\frac{2}{\pi }\leq \frac{\sin x}{x}\leq \frac{2}{\pi }+\frac{\pi -2}{\pi^{5}}\left(\pi^{4}-16x^{4}\right), \end{array}$$

for *x* ∈ (0, *π*/2]. The equalities are valid if and only if *x* = *π*/2. This also refines Jordan's inequality (1). Also, see the double inequality (3.10) in the survey article [2, page 17]. 

In recent years, the above inequalities have been refined, extended, generalized, and applied by many mathematicians in a large amount of papers. See, for example, [3–19]. For a systematic review on this topic, please refer to the expository paper [2]. 

The aim of this paper is to further refine and generalize these inequalities of Jordan type for the sine function. 

Our main results may be stated as in the following theorems.

**Theorem 1**

If *n* ≥ 0 and *m* ≥ 2 are integers, then

(4) 2m+2(2m+nπ)πm+1{(π2)m−xmexp⁡[n(x−π2)]}     +2π≤sinxx  ≤2π+2m(π−2)πm+1{(π2)m−xmexp⁡[n(x−π2)]},

on (0, *π*/2].

**Theorem 2**

Suppose that *g* is a 3-time differentiable function on [0, *π*/2]. If the function *g* satisfies *g*(*π*/2) ≠ *g*(0) and

(5) $$\begin{array}{c} g^{\prime }\left(x\right)>0,\;\;3g^{\prime \prime }\left(x\right)+xg^{\prime \prime \prime }\left(x\right)>0,\\ 2g^{\prime }\left(x\right)\leq 2xg^{\prime \prime }\left(x\right)+x^{2}g^{\prime \prime \prime }\left(x\right), \end{array}$$

on (0, *π*/2], then

(6) 4π2g′(π/2)[g(π2)−g(x)]+2π  ≤sinxx  ≤2π+π−2π[g(π/2)−g(0)][g(π2)−g(x)].

**Remark 3**

Taking *n* = 0 in Theorem 1 yields

(7) 2m+1mπm+1[(π2)m−xm]  +2π ≤sinxx ≤2π+2m(π−2)πm+1[(π2)m−xm],

on (0, *π*/2] for *m* ≥ 2. The equalities in (7) are valid if and only if *x* = *π*/2.

Putting *m* = 2,4 in (7) results in (2) and (3), respectively. This means that Theorem 1 generalizes the inequalities (2) and (3).

**Remark 4**

Let the function *g*(*x*) in Theorem 2 be *x* ^*m*^ for *m* ≥ 2. A straightforward computation gives

(8) g(π2)=(π2)m≠0=g(0),g′(x)=mxm−1>0,3g′′(x)+xg′′′(x) =3m(m−1)xm−2+xm(m−1)(m−2)xm−2>0,2g′(x)−2xg′′(x)+x2g′′′(x)=−m(m−1)(m−2)≤0,4π2g′(π/2)[g(π2)−g(x)]  =2m+1mπm+1[(π2)m−xm]+2π,π−2π[g(π/2)−g(0)][g(π2)−g(x)]  =2π+2m(π−2)πm+1[(π2)m−xm].

This implies inequality (7). Hence, Theorem 2 generalizes Theorem 1.

In the final section of this paper, we will apply Theorem 1 to refine and generalize Yang's inequality and construct some integral inequalities.

## 2. A Lemma

In order to prove Theorems 1 and 2, the following lemma is necessary.

**Lemma 5**

Let *f*, *g* : [*a*, *b*] → ℝ be differentiable on (*a*, *b*). If *g*′ ≠ 0 and *f*′/*g*′ are decreasing on (*a*, *b*), then the functions

(9) $$\begin{array}{c} \frac{f\left(x\right)-f\left(b\right)}{g\left(x\right)-g\left(b\right)},\;\;\;\frac{f\left(x\right)-f\left(a\right)}{g\left(x\right)-g\left(a\right)}, \end{array}$$

are also decreasing on (*a*, *b*).

**Remark 6**

Lemma 5 can be found in many papers such as [10, 12, 13, 20].

## 3. Proofs of Theorems

We are now in a position to prove our theorems.

**Proof of Theorem 1**

Let

(10) $$\begin{array}{c} f_{1}\left(x\right)=\frac{\sin x}{x},\;\;f_{2}\left(x\right)=-x^{m}e^{nx},\\ f_{3}\left(x\right)=\sin x-x\cos x,\;\;f_{4}\left(x\right)=\left(mx^{m+1}+nx^{m+2}\right)e^{nx}, \end{array}$$

on (0, *π*/2]. A direct calculation gives

(11) f1′(x)f2′(x)=sinx−xcos⁡x(mxm+1+nxm+2)enx=f3(x)f4(x),f3′(x)f4′(x)=sinx[m(m+1)xm−1+2n(m+1)xm+n2xm+1]enx,[f3′(x)f4′(x)]′=hm(x)sec x[m(m+1)xm−1+2n(m+1)xm+n2xm+1]2enx,

where

(12) hm(x)=m(m+1)xm−1+2n(m+1)xm+n2xm+1 −[m(m+1)(m−1)xm−2   +3nm(m+1)xm−1+3n2(m+1)xm   + n3xm+1]tanx.

Utilizing tan*x* > *x* on (0, *π*/2) leads to

(13) hm(x)≤m(m+1)xm−1+2n(m+1)xm +n2xm+1−x[m(m+1)(m−1)xm−2         +3nm(m+1)xm−1         + 3n2(m+1)xm+n3xm+1]=−[m(m+1)(m−2)xm−1+n(m+1)(3m−2)xm   + n2(3m−2)xm+1+n3xm+2]≤0,

on (0, *π*/2) for *m* ≥ 2 and *n* ≥ 0. As a result, the function *f*_3_′(*x*)/*f*_4_′(*x*) is decreasing on (0, *π*/2). In virtue of Lemma 5, it follows that the functions

(14) $$\begin{array}{c} \frac{f_{3}\left(x\right)}{f_{4}\left(x\right)}=\frac{f_{3}\left(x\right)-f_{3}\left(0\right)}{f_{4}\left(x\right)-f_{4}\left(0\right)},\;\;\frac{f_{1}^{\prime }\left(x\right)}{f_{2}^{\prime }\left(x\right)},\\ H\left(x\right)=\frac{f_{1}\left(x\right)-f_{1}\left(\pi /2\right)}{f_{2}\left(x\right)-f_{2}\left(\pi /2\right)} \end{array}$$

are all decreasing on (0, *π*/2). Since

(15) $$\begin{array}{c} \underset{x\to 0^{+}}{\lim}H\left(x\right)=\frac{2^{m}\left(\pi -2\right)}{\pi^{m+1}}e^{-n\pi /2},\\ \underset{x\to {\left(\pi /2\right)}^{-}}{\lim}H\left(x\right)=\frac{2^{m+2}}{\left(2m+n\pi \right)\pi^{m+1}}e^{-n\pi /2}, \end{array}$$

we have

(16) $$\begin{array}{c} \frac{2^{m+2}}{\left(2m+n\pi \right)\pi^{m+1}}e^{-n\pi /2}\leq H\left(x\right)\leq \frac{2^{m}\left(\pi -2\right)}{\pi^{m+1}}e^{-n\pi /2}, \end{array}$$

which can be reformulated as the inequality (4). Theorem 1 is thus proved.

**Proof of Theorem 2**

Let

(17) $$\begin{array}{c} f_{1}\left(x\right)=\frac{\sin x}{x},\;\;f_{2}\left(x\right)=-g\left(x\right),\\ f_{3}\left(x\right)=\sin x-x\cos x,\;\;f_{4}\left(x\right)=x^{2}g^{\prime }\left(x\right), \end{array}$$

on (0, *π*/2]. It is easy to see that

(18) f2′(x)=−g′(x)<0,  f2′(x)≠0,f4′(x)≠0,f4′(x)=2xg′(x)+x2g′′(x)=x(2g′(x)+xg′′(x))>0,

on (0, *π*/2]. Furthermore, we have

(19)f1′(x)f2′(x)=sinx−xcos⁡xx2g′(x)=f3(x)f4(x),f3′(x)f4′(x)=sinx2g′(x)+xg′′(x),(20)[f3′(x)f4′(x)]′=2g′(x)+xg′′(x)−[3g′′(x)+xg′′′(x)]tanx[2g′(x)+xg′′(x)]2secx.

Employing tan*x* > *x* and the conditions in (5), it is not difficult to show that the numerator of [*f*_3_′(*x*)/*f*_4_′(*x*)]′ is negative on (0, *π*/2). This means that the function *f*_3_′(*x*)/*f*_4_′(*x*) is decreasing on (0, *π*/2). Consequently, making use of Lemma 5 consecutively, it is revealed that the functions

(21) $$\begin{array}{c} \frac{f_{3}\left(x\right)}{f_{4}\left(x\right)}=\frac{f_{3}\left(x\right)-f_{3}\left(0\right)}{f_{4}\left(x\right)-f_{4}\left(0\right)},\;\;\frac{f_{1}^{\prime }\left(x\right)}{f_{2}^{\prime }\left(x\right)},\\ H\left(x\right)=\frac{f_{1}\left(x\right)-f_{1}\left(\pi /2\right)}{f_{2}\left(x\right)-f_{2}\left(\pi /2\right)}=\frac{\left(\sin x/x\right)-\left(2/\pi \right)}{g\left(\pi /2\right)-g\left(x\right)} \end{array}$$

are all decreasing on (0, *π*/2). Since

(22) lim⁡x→0+H(x)=lim⁡x→0+f1(x)−f1(π/2)f2(x)−f2(π/2)=1−(2/π)g(π/2)−g(0)=π−2π[g(π/2)−g(0)],lim⁡x→(π/2)−H(x)=lim⁡x→(π/2)−((sinx)/x)−(2/π)g(π/2)−g(x)=lim⁡x→(π/2)−sinx−(2/π)xxg(π/2)−xg(x)=lim⁡x→(π/2)−cos⁡x−(2/π)g(π/2)−g(x)−xg′(x)=−(2/π)−(π/2)g′(π/2)=4π2g′(π/2),

from *H*(*π*/2) ≤ *H*(*x*) ≤ *H*(0), the inequality (6) follows. The proof of Theorem 2 is complete.

## 4. Applications of Theorem 1

After proving Theorems 1 and 2, we now start off to apply them to construct some new inequalities. 

Let 0 ≤ *λ* ≤ 1 and *A*, *B* > 0 with *A* + *B* ≤ *π*. Then,

(23) cos⁡2(λA)+cos⁡2(λB)−2cos⁡(λA)cos⁡(λB)cos⁡(λπ)  ≥sin2(λπ).

This inequality is known in the literature as Yang's inequality. Since paper [16], many mathematicians mistakenly referred this inequality to [21, pages 116–118]. Indeed, the paper we should refer to is [22] or an even earlier paper in Chinese.

The first application of Theorem 1 is to refine and generalize Yang's inequality (23) as follows.

**Theorem 7**

For *k* ≥ 2, let *A*_*i*_ > 0 and ∑_*i*=1_^*k*^ *A*_*i*_ ≤ *π*. If 0 ≤ *λ* ≤ 1, then

(24) $$\begin{array}{c} R\left(\lambda \right)\leq \sum_{1\leq i<j\leq k}H_{ij}\leq T\left(\lambda \right), \end{array}$$

where

(25) Hij=cos⁡2(λAi)+cos⁡2(λAj) −2cos⁡(λAi)cos⁡(λAj)cos⁡(λπ),T(λ)=4(k2){λ+(π−2)λ2[1−λmenπ(λ−1)/2]}2  ,R(λ)=4(k2){λ+2λ2m+nπ[1−λmenπ(λ−1)/2]}2cos⁡2λπ2,

and *n* ≥ 0 and *m* ≥ 2 are integers.

**Proof**

Substituting *x* = *λπ*/2 in the inequality (4) reveals that

(26) $$\begin{array}{c} \sin\frac{\lambda \pi }{2}\geq \lambda +\frac{2\lambda }{2m+n\pi }\left[1-\lambda^{m}e^{n\pi /2(\lambda -1)}\right],\\ \sin\frac{\lambda \pi }{2}\leq \lambda +\frac{\left(\pi -2\right)\lambda }{2}\left[1-\lambda^{m}e^{n\pi /2(\lambda -1)}\right]. \end{array}$$

Using

(27) $$\begin{array}{c} \sin^{2}\left(\lambda \pi \right)=4\sin^{2}\frac{\lambda \pi }{2}\cos^{2}\frac{\lambda \pi }{2}, \end{array}$$

the inequality

(28) $$\begin{array}{c} \sin^{2}\left(\lambda \pi \right)\leq H_{ij}\leq 4\sin^{2}\frac{\lambda \pi }{2}, \end{array}$$

see either [22], [16, (2.13)], or [2, page 17, (3.4)], becomes

(29) 4{λ+2λ2m+nπ[1−λmenπ/2(λ−1)]}2cos⁡2λπ2≤Hij  ≤4{λ+(π−2)λ2[1−λmenπ/2(λ−1)]}2.

Finally, taking the sum of the above inequality for all 1 ≤ *i* < *j* ≤ *n* results in (24). The required proof is complete.

**Corollary 8**

Under the conditions of Theorem 7, one has

(30) $$\begin{array}{c} R_{1}\left(\lambda \right)\leq \sum_{1\leq i<j\leq k}H_{ij}\leq T_{1}\left(\lambda \right), \end{array}$$

where

(31) $$\begin{array}{c} T_{1}\left(\lambda \right)=\left(\begin{array}{c} k\\ 2 \end{array}\right)\pi^{2}\lambda^{2},\;\;R_{1}\left(\lambda \right)=4\left(\begin{array}{c} k\\ 2 \end{array}\right)\lambda^{2}\cos^{2}\frac{\lambda \pi }{2}. \end{array}$$

**Proof**

When 0 ≤ *λ* < 1 and *n* = 0, we have

(32) $$\begin{array}{c} \underset{m\to \infty }{\lim}T\left(\lambda \right)=\left(\begin{array}{c} k\\ 2 \end{array}\right)\pi^{2}\lambda^{2},\;\;\underset{m\to \infty }{\lim}R\left(\lambda \right)=4\left(\begin{array}{c} k\\ 2 \end{array}\right)\lambda^{2}\cos^{2}\frac{\lambda \pi }{2}. \end{array}$$

This implies the required result.

The second application of Theorem 1 is to construct some new integral inequalities for sin*x*/*x*.

**Theorem 9**

For *x* ∈ (0, *π*/2], if *n* ≥ 0 and *m* ≥ 2 are integers, then

(33) ∫0π/2sinxxdx+2m+2e−nπ/2(2m+nπ)πm+1∫0π/2xmenxdx  ≥1+2(2m+nπ),∫0π/2sinxxdx+2m(π−2)e−nπ/2πm+1∫0π/2xmenxdx  ≤π2.

**Proof**

This follows from integrating on all sides of the double inequality (4).

**Remark 10**

Applying Theorem 9 to *n* = 0 gives

(34) $$\begin{array}{c} \frac{1}{m+1}+1\leq \int_{0}^{\pi /2}\frac{\sin x}{x}dx\leq 1+\frac{\left(\pi -2\right)}{2}\frac{m}{m+1}. \end{array}$$

Applying Theorem 9 to *n* = 0 and *m* = 2 yields

(35) $$\begin{array}{c} \frac{4}{3}\leq \int_{0}^{\pi /2}\frac{\sin x}{x}dx\leq \frac{\pi +1}{3}. \end{array}$$

This is a recovery of an inequality established in [3, page 101]. It was also collected in [2, (2.14)]. Such a kind of inequalities can be found in [23].
